# Supplementary material for: A Global Screen for Assembly State Changes of the Mitotic Proteome by SEC-SWATH-MS
Source: Cell Syst. 2020 Feb 26;10(2):133–155.e6. doi: 10.1016/j.cels.2020.01.001 (PMC7042714; doi:10.1016/j.cels.2020.01.001)

Q04637 | IF4G1_HUMAN | EIF4G1 EIF4F EIF4G EIF4GI
Monomer MW [kDa]: 175.491 Monomer expected elution fraction: 35

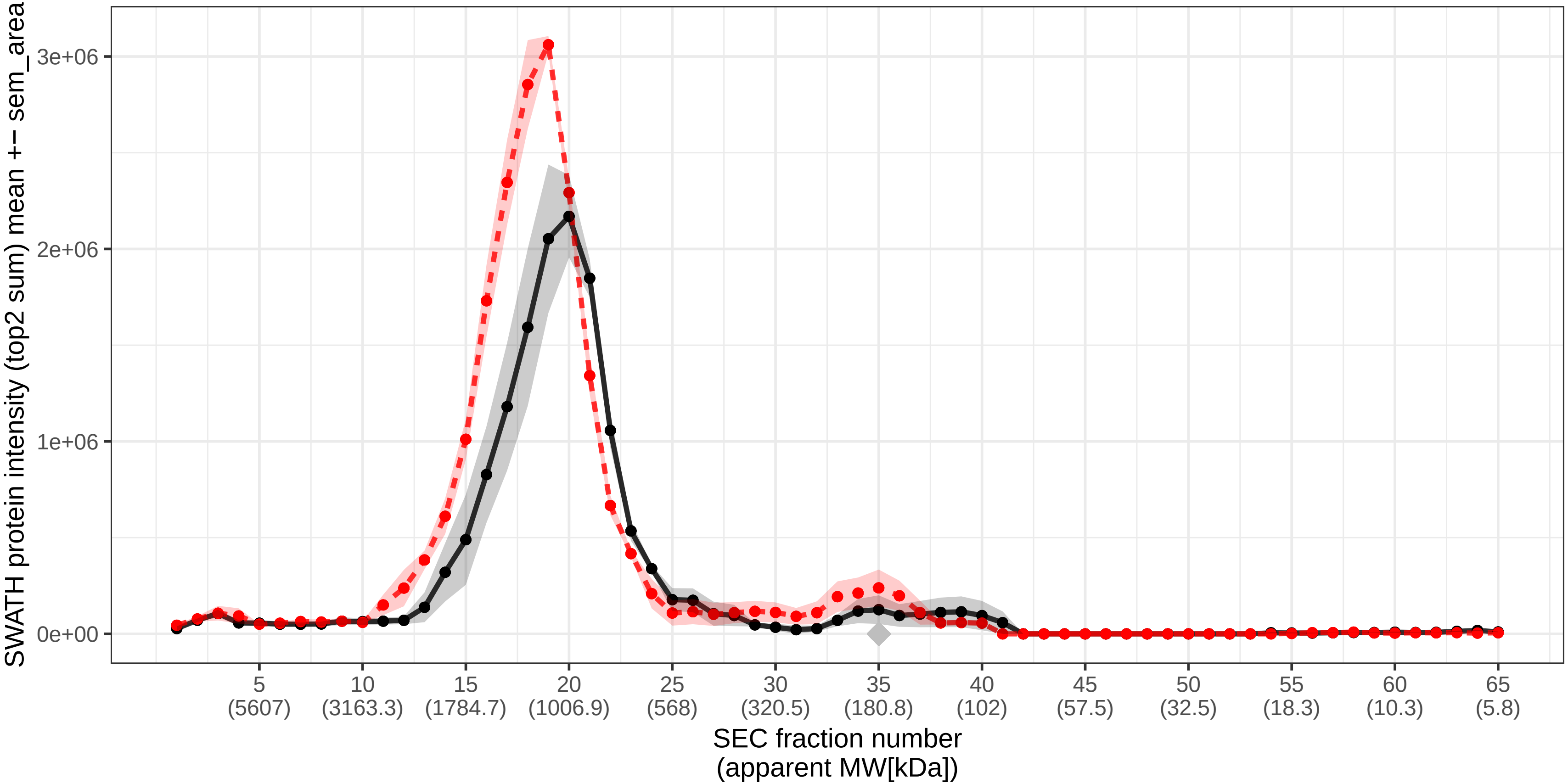

Supplement: Data S1. SEC-SWATH-MS Protein Chromatograms, Related to Figure 1 [file mmc6.zip › SECchrom_Q04637_IF4G1_HUMAN_EIF4G1_EIF4F_EIF4G_EIF4GI.pdf]
